# Force Measurement Enabling Precise Analysis by Dynamic Force Spectroscopy

**DOI:** 10.3390/ijms13010453

**Published:** 2011-12-29

**Authors:** Atsushi Taninaka, Yuuichi Hirano, Osamu Takeuchi, Hidemi Shigekawa

**Affiliations:** Institute of Applied Physics, University of Tsukuba, 1-1-1 Tennodai, Tsukuba 305-8573, Japan; E-Mails: jun_t@bk.tsukuba.ac.jp (A.T.); bk201120358@s.bk.tsukuba.ac.jp (Y.H.); takeuchi@bk.tsukuba.ac.jp (O.T.)

**Keywords:** dynamic force spectroscopy, atomic force microscopy, potential landscape, functional molecules, molecular recognition

## Abstract

Dynamic force spectroscopy (DFS) makes it possible to investigate specific interactions between two molecules such as ligand-receptor pairs at the single-molecule level. In the DFS method based on the Bell-Evans model, the unbinding force applied to a molecular bond is increased at a constant rate, and the force required to rupture the molecular bond is measured. By analyzing the relationship between the modal rupture force and the logarithm of the loading rate, microscopic potential barrier landscapes and the lifetimes of bonds can be obtained. However, the results obtained, for example, in the case of streptavidin/biotin complexes, have differed among previous studies and some results have been inconsistent with theoretical predictions. In this study, using an atomic force microscopy technique that enables the precise analysis of molecular interactions on the basis of DFS, we investigated the effect of the sampling rate on DFS analysis. The shape of rupture force histograms, for example, was significantly deformed at a sampling rate of 1 kHz in comparison with that of histograms obtained at 100 kHz, indicating the fundamental importance of ensuring suitable experimental conditions for further advances in the DFS method.

## 1. Introduction

The various interactions between a pair of functional molecules, for example, DNA, ligand-receptor and antigen-antibody systems, play essential roles in biological processes and molecular devices based on molecular recognition properties [1–9]. Therefore, to further understand molecular recognition properties and apply them in functional devices, it is of great importance to analyze the reaction processes of molecules with respect to a variety of local structures and their properties. Despite this importance, however, local variations between two molecules involved in chemical reactions might not have been well probed by conventional techniques that obtain averaged information [10,11].

Dynamic force spectroscopy (DFS) is a technique that enables the investigation of specific interactions between two molecules at the single-molecule level [5–9,12–31]. In the DFS method based on the Bell-Evans model [13,14], the unbinding force applied to a pair of molecules is increased at a constant rate, and the force required to rupture the bond is measured. When an unbinding force *f* is applied to a pair of molecules, the height of the potential barrier *E* is reduced to *E* − *fx*_b_, where *x*_b_ is the potential barrier position. Therefore, the rupture probability depends on the magnitude of the applied force. Using the Bell-Evans model [13,14], the relationship between the modal rupture force and the loading rate is stochastically analyzed, and thereby, the potential barrier position *x*_b_ and the bond lifetime are obtained. There are no other techniques that enable the analysis of chemical reaction processes at the single-molecule level, and DFS has been successfully used to analyze the molecular dynamics of systems such as ligand-receptor pairs and DNA molecules [5–9,12–31].

Several modifications of the original Bell-Evans model [13,14] have been proposed and advanced methods of analysis have been developed during the past few decades [17–23]. The rupture processes based on the wormlike chain model or the unzipping model that treats such as unraveling of DNA has been discussed and the effect processes such as the rebinding process has been clarified using these models [17–23]. However, even when a system without the rebinding process is analyzed, for example, interactions between streptavidin/avidin and biotin molecules, the obtained results for potential barriers and bond lifetimes differ among studies, and some results have been inconsistent with theoretical predictions [13–23]. By considering the results of previous DFS experiments, the significance of the effect of experimental conditions such as the sample preparation method on experimental results has been clarified in addition to the issue of the loading rate [28–31].

In this paper, we further analyze the experimental conditions; we investigated the effect of the sampling rate on DFS measurement using an avidin-biotin complex. In DFS analysis based on the Bell-Evans model [13,14], it is necessary to produce a histogram of the rupture force observed at a constant loading rate. The loading rate must also be varied between 10 pN/s and 10^5^ pN/s [28–31]. However, the sampling rate of 1 kHz is not sufficiently high to measure the rapid changes in the force curve at a large loading rate. Moreover, for a small loading rate, since the rupture force is as small as the force induced by the thermal excitation of the AFM cantilever, it is also difficult to determine the rupture force at such a low sampling rate. These issues make it difficult to acquire accurate rupture forces at both large and small loading rates, resulting in the observed variations in the potential barrier positions and bond lifetimes.

These points must be considered before discussing the details of molecular interactions. In fact, although such effects have not been examined in detail, we found that the sampling rate of 1 kHz has a critical effect on rupture-force measurement. For a low loading rate, in particular, the shape of rupture-force histograms obtained at a 1 kHz sampling rate was significantly deformed in comparison with that of histograms obtained at 100 kHz, indicating the fundamental importance of suitable experimental conditions when employing the DFS method.

## 2. Theory and Experimental Methods

**Figure 1:**
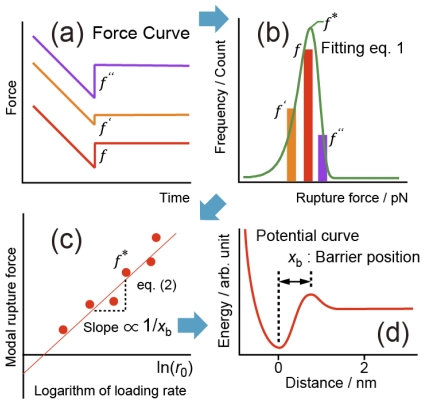
Schematic diagrams to explain the analysis method of Dynamic force spectroscopy (DFS). (**a**) Force curves; (**b**) Rupture force histogram. The modal rupture force is the most frequent rupture force [14] determined for each loading-rate measurement by fitting the rupture-force histogram with equation 1; (**c**) Modal rupture force as a function of loading rate; (**d**) Potential landscape.

First, we explain the methodology that we developed and the details of sample preparation. Figure 1 shows schematic diagrams to explain the analysis method of DFS [13,14,24–31]. If an unbinding force *f* is applied to a pair of molecules, the height of the potential barrier *E* is reduced to *E* − *fx*_b_. Since the lifetime of a molecular bond depends on the potential barrier height, the rupture probability depends on the magnitude of the applied force [13,14,31]. In DFS, the unbinding force applied to a pair of molecules is increased at a constant rate, and the force required to rupture the bond is measured. According to the Bell-Evans model [13,14], when a tensile force is applied at a constant loading rate, the probability distribution of the rupture force can be expressed as

(1) $$P_{\left(f\right)}=Cexp\{(f-f^{*})x_{b}/k_{B}T\}exp[1-exp\{(f-f^{*})x_{b}/k_{B}\;T\}]$$

where *P*_(_*_f_*_)_, *f*, f^*^, *x*_b_, *k*_B_, *T* and *C* are the probability distribution of the rupture force, the rupture force, the most frequent rupture force, the distance of the potential barrier position from the potential bottom, the Boltzmann constant, the temperature and a normalization constant, respectively [13,14,28–31]. The modal rupture force is the most frequent rupture force [14] determined for each loading-rate measurement by fitting the rupture-force histogram with equation 1 as shown in Figure 1(b).

According to equation 1,f^*^ linearly depends on the logarithm of the loading rate *r*_0_ (d*f*/d*t*),

(2) $$f^{*}=k_{B}T/x_{b}\{\text{ln }r_{0}+\text{ln}(t_{off(0)}x_{b}/k_{B}\;T)\}$$

where *t*_off(0)_ is the lifetime of the molecular bond. Equation 2 indicates that the potential barrier position can be obtained from the slope of the linear relationship (Figure 1(c)) [13,14,28–31].

To enable the use of equations 1 and 2, it is essential to maintain a constant loading rate [13,31]. However, when DFS measurement is carried out using an AFM with a cross-linker molecule, a constant retraction velocity does not result in a constant loading rate because of the stretching of the cross-linker molecule [17,18]. In addition, the observed force in DFS may include viscous drag acting on the cantilever. Since a constant loading rate has been achieved in previous experiments, such an effect can be taken into consideration [29]. Therefore, we introduce a mechanism for controlling the applied force to maintain a constant loading rate. We have developed an AFM system with a force feedback mechanism that enables precise analysis by DFS [28–31], which features the fine control of the loading rate to reduce the effect of a soft cross-linker molecule connecting a sample molecule to the tip or substrate [28–31].

As shown in Figure 1, for a selected loading rate, many rupture forces can be measured from force curves [24,25]. The measured rupture forces are shown in a histogram, from which the modal rupture force is obtained for each loading rate. The modal rupture force is the most frequent rupture force f^*^ [14] determined for each loading-rate measurement by fitting the histogram with equation 1 as shown in Figure 1(b). In the analysis, the modal rupture forces are plotted as a function of the logarithm of the loading rate, thereby, information concerning the energy landscape of the interaction is derived from the relationship between the modal rupture force obtained from the histogram and the loading rate of the unbinding force.

Force curves were measured at a high sampling rate (100 kHz) for various constant loading rates, and then these force curves were numerically converted to those at lower sampling rates of 10 kHz and 1 kHz. Namely, every 10 and every 100 data were picked up from the full series of the original data obtained for the 100 kHz measurement and treated on the basis of the sampling theorem to provide the data for 10 kHz and 1 kHz, respectively. The method of resampling that we carried out is based on the sampling theorem (Nyquist’s theorem). The data obtained at 100 kHz is sufficient to produce sampling rates of 10 kHz and 1 kHz. To reduce the sampling rate, for example, from 100 kHz to 10 kHz, the data obtained at 100 kHz was changed after the application of a low-pass filter with a frequency of 5 kHz. The resampling process is considered sufficient to reduce the amount of data to that actually observed at a small sampling rate.

**Figure 2:**
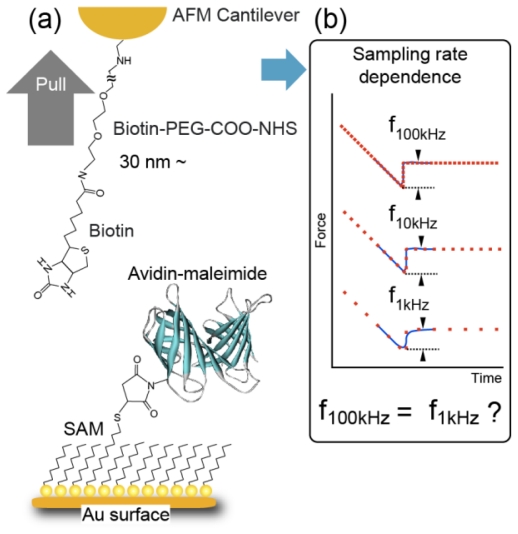
Schematic illustrations of the method of sample preparation and the effect of sampling rate on force curve measurement.

As described in the introduction, in this study, we focused on the sampling rate used in force measurement. This is because, as shown in Figure 2(b), if the sampling rate is not sufficiently high, the rupture point in a force curve cannot be determined accurately. For a low loading rate, the measured rupture force is smaller than the actual rupture force. For a high loading rate, the number of measurement points is fewer and it becomes difficult to measure the rupture force accurately.

Figure 2(a) shows schematic illustrations of the method of sample preparation. We used an avidin-biotin complex, a typical ligand-receptor system, as a sample [10,11]. Avidin-maleimide was synthesized using avidin molecules (1 mg/mL in phosphate-buffered saline (PBS)) and 0.1 mg/mL of sulfo-SMCC, and the activated avidin was purified by desalting on a column and by high-performance liquid chromatography (HPLC). First, as a substrate, a thin gold film (100 nm) was evaporated onto a freshly cleaved mica surface in a high vacuum at 400 °C and flame-annealed using a hydrogen gas burner for 30 s. Then, the substrate was immersed in a solution of 1, 10-decanedithiol/1-octanethiol (1/100 ratio) (1 mM in ethanol) for 48 h to form a closely packed self-assembled monolayer (SAM) with thiol groups on the surface. The substrate was then immersed in a solution of avidin-maleimide for 5 h to fix avidin molecules to the substrate. The substrates were washed in PBS several times after the immobilization of avidin molecules.

A gold-coated cantilever was immersed in a solution of 8-amino, 1-octanethiol hydrochloride molecules (1 mM in ethanol) for 48 h to form a closely packed SAM with amino groups on the surface. After rinsing with ethanol, the cantilever was immersed in a solution of biotin-PEG3400-COO-NHS molecules (Shearwater Polymers, 0.1 mM in ethanol) for 20 h to fix a biotin (biotin-PEG) molecule onto the probe apex. Finally, the cantilever was rinsed with ethanol. To investigate the effect of the sampling rate, Bio-Levers (Bio-Lever, Olympus, 0.006 N/m and 0.03 N/m, rectangular shape) and microcantilevers (OMCL-RC800PB, Olympus, 0.06 N/m, rectangular shape) were prepared. These cantilevers were chosen because their stiffness is typical of cantilevers widely used in DFS experiments using an AFM. The soft cantilever of 0.006 N/m stiffness is suitable for obtaining a weak force in a small-loading rate measurement, while the cantilevers of 0.06 N/m and 0.03 N/m stiffness are suitable for measuring a stronger force a large-loading rate measurement.

To avoid multiple-bonding events, the density of the target molecules in the SAM was reduced and free biotin molecules were introduced so that the probability of bonding became 5–10% for each tip-sample approach. Only one peak becomes dominant after this treatment [29,32–36]. Furthermore, only single ruptures were counted to remove errors caused by the effect of multiple-rupture events on the analysis. We used a 0.05 M sodium nitrate solution (pH 7) for DFS measurements, in which the potential barrier for the avidin-biotin interaction was estimated to be at 0.3 nm [13,14,30]. All DFS measurements were performed at room temperature.

## 3. Results and Discussion

**Figure 3:**
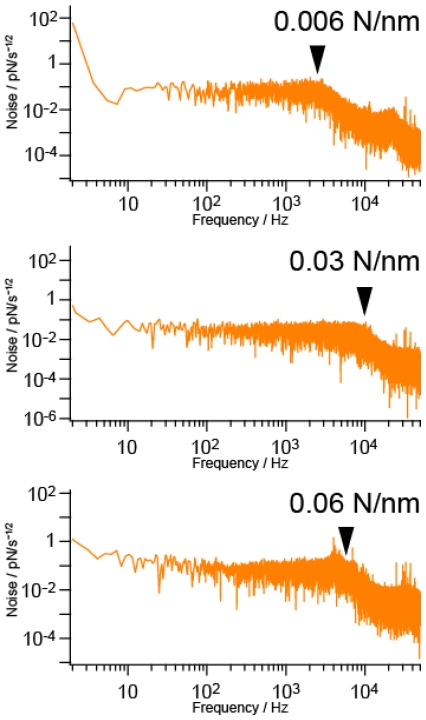
Power spectra of the three cantilevers with spring constants of 0.006 N/m, 0.03 N/m and 0.06 N/m, whose cutoff frequencies were estimated to be 2 kHz, 10 kHz and 5 kHz, respectively.

Figure 3 shows the power spectra of the three cantilevers with spring constants of 0.006 N/m, 0.03 N/m and 0.06 N/m, whose cutoff frequencies were estimated to be 2 kHz, 10 kHz and 5 kHz, respectively, as indicated by arrows. The resonance peak in each spectrum is small and not clear due to the effect of the solution in which the cantilevers are placed.

To clarify and remove the effect of noise on measurement when the signal level is low, the sampling rate should be 5–10 times larger than the cutoff frequencies indicated by arrows in Figure 3. Namely, the sampling rate should be larger than 10 kHz, 50 kHz and 30 kHz for the cantilevers with spring constants of 0.006 N/m, 0.03 N/m and 0.06 N/m, respectively. However, even in the case of a low signal to noise (S/N) ratio, a typical sampling rate used for measurement using a commercial AFM has been 1 kHz. Therefore, it is of great importance to clarify the effect of sampling rate on measurement. For this purpose, we compared the results obtained at 1 kHz, 10 kHz and 100 kHz.

**Figure 4:**
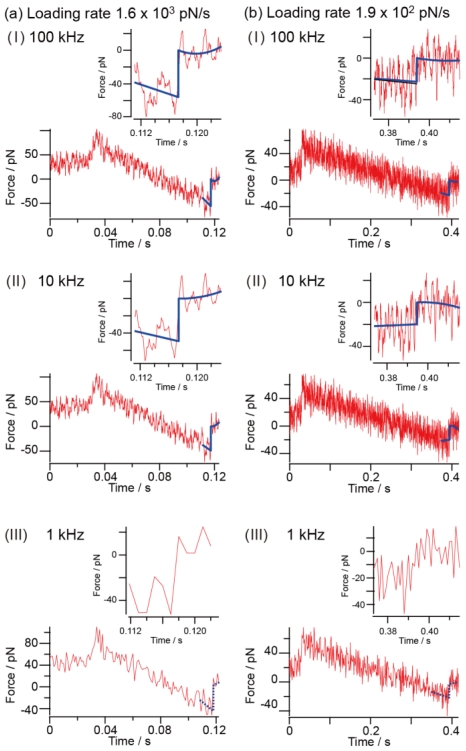
Force curves obtained at a sampling rate of 100 kHz for loading rates of 1.6 × 10^3^ pN/s ((a)-I to (a)-III) and 1.9 × 10^2^ pN/s ((b)-I to (b)-III), where force curves for 10 kHz and 1 kHz were numerically converted from data obtained at a sampling rate of 100 kHz.

Figure 4 shows force curves obtained at a sampling rate of 100 kHz for loading rates of 1.6 × 10^3^ pN/s (4(a)-I to 4(a)-III) and 1.9 × 10^2^ pN/s (4(b)-I to 4(b)-III), where the force curves for 10 kHz and 1 kHz were numerically obtained, as described above, from the data obtained at a sampling rate of 100 kHz. The method of obtaining the rupture force is similar to that by Kasas *et al*. [24]. The rupture point corresponds to a peak value of the derivative of the force curve. We estimate the rupture point in the graph from the peak position, and by fitting the force curves before and after the rupture point, we can evaluate the rupture force. Although the determination of the rupture point is essential to estimate the rupture force, if the sampling rate is not sufficient, it is difficult to determine as explained below.

For the loading rate of 1.6 × 10^3^ pN/s (Figure 4(a)), since rupture occurs at a larger force, the S/N ratio is sufficiently high. However, since the force changes more rapidly than in the case of a loading rate of 1.9 × 10^2^ pN/s, the smaller number of measurement points becomes a critical issue. Although the rupture force is clearly determined for sampling rates of 10 kHz and 100 kHz, it cannot be accurately measured for the sampling rate of 1 kHz owing to the reduced number of measurement points (10 measurement points in this case).

For the loading rate of 1.9 × 10^2^ pN/s, since the magnitude of the rupture force becomes close to that of the thermal noise, we should carefully choose an appropriate sampling rate. A rupture force of about 20 pN was observed for sampling rates of 100 kHz and 10 kH. However, if the sampling rate is insufficient, the differential coefficient used to obtain the position of the rupture in the force curve cannot be determined with confidence under thermal noise. In fact, as shown in Figure 4(b)-III, the rupture force tends to be determined smaller for the sampling rate of 1 kHz. To remove the effect of thermal noise, a much higher sampling rate should be used.

How high should the sampling rate be necessary for measurement? In DFS, as explained concerning the procedures shown in Figure 1, many rupture forces are measured from the force curves for a selected loading rate. The measured rupture forces are summarized in a histogram, from which the modal rupture force is obtained for the loading rate. After repeating the procedure for various loading rates, the obtained modal rupture forces are plotted as a function of the logarithm of the loading rate, thereby, information concerning the energy landscape of the interaction, the potential barrier position and the lifetime is derived. The loading rate, in general, must be varied from a very small value of 10 pN/s to a large value of 10^5^ pN/s. A higher sampling rate is necessary for measurement at a larger loading rate. For the examples shown in Figure 4(a), the loading rate is 10^3^ pN/s. To obtain an accurate rupture force at a loading rate of 10^5^ pN/s, it is necessary to observe the force curve obtained at a significantly higher sampling rate. For example, when the cantilever with a spring constant of 0.06 N/m is used at a sampling rate of 20 kHz, about 200 measurement points are obtained even at a loading rate of 10^5^ pN/s, enabling the accurate estimation of the rupture force.

**Figure 5:**
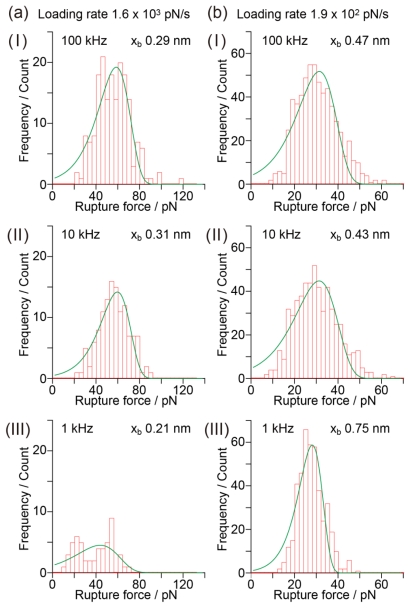
Histograms of the rupture force obtained for sampling rates of 100 kHz, 10 kHz and 1 kHz based on a series of data shown in Figure 4.

How does the measurement inaccuracy affect the histogram? As has been discussed, the rupture force cannot be determined when the sampling rate used in the force curve measurement is not sufficiently high; this affects the analysis of the histogram. Figure 5 shows histograms of the rupture force obtained for sampling rates of 100 kHz, 10 kHz and 1 kHz based on a series of data shown in Figure 4. The histogram for 10 kHz has a similar shape to that for 100 kHz. In contrast, the histogram for 1 kHz is deformed and different from those for 100 kHz and 10 kHz. In general, the width of a histogram showing the distribution of rupture forces is considered to increase when there is an experimental error in the force measurement. In the case shown in Figure 5, however, the width of the histogram decreases as the sampling rate becomes low. Since the experimental error increases with decreasing sampling rate, the obtained results indicate that the rupture forces have not been accurately estimated.

**Figure 6:**
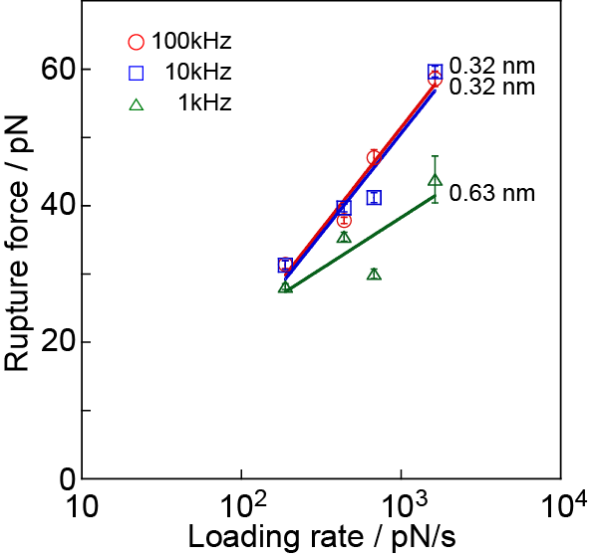
Relationship between the modal rupture forces and the logarithm of the loading rate at sampling rates of 100 kHz, 10 kHz and 1 kHz, which correspond to the values derived from the histograms shown in Figure 5.

This inaccuracy affects the final result, *i.e.*, the distance of the potential barrier position. Since the modal rupture force is obtained by fitting equation 1 to the histograms obtained by changing the loading rate, the modal rupture force changes with the sampling rate. Figure 6 shows the relationship between the modal rupture forces and the logarithm of the loading rate at sampling rates of 100 kHz, 10 kHz and 1 kHz, which correspond to the values derived from the histograms shown in Figure 5. Despite the fact that the data for 1 kHz and 10 kHz are derived from the same series of data, *i.e.*, data measured at 100 kHz, the slope of the line, which provides the potential barrier position, is modified for the sampling rate of 1 kHz (0.63 ± 0.30 nm for 1 kHz, 0.32 ± 0.06 nm for 10 kHz, and 0.32 ± 0.04 nm for 100 kHz). This result indicates that an insufficient sampling rate causes a significant error in the analysis. The difference in the magnitude of forces changes the lifetime, which is strongly affected by the measurement conditions compared with their effect on the potential barrier positions. To clarify the variation in lifetimes, further analysis is necessary.

There is another method by which the potential barrier position can be estimated by analyzing the shape of the rupture force distribution [28–31]. Using this method, the potential barrier position can be estimated from a single histogram. This means that the experimental efficiency is markedly improved. Namely, 3000–10,000 approach and retract cycles are carried out to form a histogram for each loading rate measurement in general DFS measurement [5–9,12–31]; thus, it takes a long time to form the line in Figure 1, from which the potential barrier position and bond lifetime are derived. In contrast, with the new method, we can obtain the result from a single histogram.

Table 1 shows the potential barrier position estimated by the analysis of the shape of the rupture force distribution for each sampling rate. To discuss the shape of the histogram and to estimate the potential barrier position by analyzing the shape of the rupture force distribution, it is necessary to obtain a precise force curve at a sufficiently large sampling rate and at a constant loading rate. In Figure 6, the histogram for 1 kHz appears to be well fitted by equation 2. However, the potential barrier position estimated by analyzing the shape of the rupture force histogram for 1 kHz is 0.75 nm, which does not correspond to 0.32 nm, the accurate value obtained from the slope in Figure 6. When the loading rate is low, it is difficult to detect a small rupture force from a force curve, which decreases the number of small rupture forces in the histogram. In such a case, it becomes difficult to accurately fit the histogram using equation 1, resulting in the variation of *x*_b_ in Table 1.

This result indicates that although the method used here has very high efficiency, when the potential position is estimated from the peak width, it is extremely important to measure the force curves at a sufficiently high sampling rate.

## 4. Conclusions

Using an atomic force microscopy technique that enables the precise analysis of molecular interactions on the basis of DFS, we investigated the effect of the sampling rate on DFS analysis. The sampling rate of 1 kHz is not sufficiently high at both large and small loading rates, resulting in a difference in the slope of the relationship between the modal rupture force and the logarithm of the loading rate, which is used to obtain potential barrier positions and bond lifetimes. The potential barrier position can be estimated from a single histogram when rupture forces are accurately measured. However, the shape of rupture force histograms was found to be significantly deformed at a sampling rate of 1 kHz in comparison with that of histograms obtained at 100 kHz, indicating the fundamental importance of suitable experimental conditions for further advances in the DFS method. The consideration of suitable experimental conditions, which have not been well investigated, is essential for increasing the usefulness and practicality of DFS analysis.

## Figures and Tables

**Table 1:** Potential barrier position estimated from the distribution width, *i.e.*, analysis of the shape of the rupture force distribution for each sampling rate.

Loading rate	*x*_b_ (100 kHz)	*x*_b_ (10 kHz)	*x*_b_ (1 kHz)
1.6 × 10^3^ pN/s	0.29 nm	0.31 nm	0.21 nm
6.8 × 10^2^ pN/s	0.30 nm	0.42 nm	0.62 nm
4.4 × 10^2^ pN/s	0.57 nm	0.51 nm	0.54 nm
1.9 × 10^2^ pN/s	0.47 nm	0.43 nm	0.75 nm
